# Assessment of the oxidative stress intensity and the integrity of cell membranes under the manganese nanoparticles toxicity in wheat seedlings

**DOI:** 10.1038/s41598-024-53697-7

**Published:** 2024-02-07

**Authors:** Apolonia Sieprawska, Elżbieta Rudolphi-Szydło, Magdalena Skórka, Anna Telk, Maria Filek

**Affiliations:** 1 Department of Biochemistry and Biophysics, Institute of Biology, University of the National Education Commission, Podchorążych 2, 30-084 Krakow, Poland; 2 Department of Analytical Chemistry, Faculty of Chemistry, Jagiellonian University, Gronostajowa 2, 30-387 Krakow, Poland

**Keywords:** Biochemistry, Biophysics, Plant sciences, Environmental sciences

## Abstract

A response to manganese nanoparticles was studied in seedlings of two wheat cultivars and a model system of plant cell membranes. Nanoparticles at concentrations of 125 and 250 mg/ml were applied foliar. The application of NPs enhanced the content of Mn in plant cells, indicating its penetration through the leaf surface. The stressful effect in the plant cells was estimated based on changes in the activity of antioxidant enzymes, content of chlorophylls and starch. MnNPs evoked no significant changes in the leaf morphology, however, an increase in enzyme activity, starch accumulation, and a decrease in chlorophyll synthesis indicated the stress occurrence. Moreover, a rise in the electrokinetic potential of the chloroplast membrane surface and the reconstruction of their hydrophobic parts toward an increase in fatty acid saturation was found.

## Introduction

As manganese (Mn) in micro amounts is necessary for proper growth and development of plants, knowledge on its reactions in biological systems is important for growing sustainable and ecological crops. Both deficiency and excess of this element in the environment distorts the course of physiological processes in plant cells. Relatively little is known about negative effects of Mn even though many papers indicated increasing soil contamination with its compounds^1^. Moreover, a potential intensification of Mn concentration in the environment results from over-fertilization and climate change^2^. Drought and hot summers favor soil acidification influence on the increasing content of Mn(II), i.e. the form in which the element is the most easily absorbed by the plant root system^3,4^. From the roots, Mn is transported to the aerial parts and accumulated in the leaves^5^. Excessive accumulation of Mn in chloroplasts distorts their structure and disrupts cell performance due to initiation of oxidative stress^6^.

The fact that metals and their oxides in the form of nanoparticles (NPs), applied at lower concentrations than their ionic counterparts, initiate similar physiological responses indicates that by using NPs in fertilizers it may be possible to reduce the increasing excess of unfavorable elements in the environment. Studies by Pradhan et al.^7,8^, showing that both in vitro and in vivo treatment with MnNPs increased photophosphorylation of chloroplasts and stimulated nitrogen metabolism suggested their use as potential nano-modulators for improving plant productivity. However, the same as for ionic forms, the effects of micronutrients provided in the form of nanoparticles depend on their concentration^9^. Hence, the excess of MnNPs in the cells can also disturb functioning of many biochemical systems.

For plants, their cell walls may serve as a barrier for the introducing of NPs, especially those of greater dimensions^10,11^. NPs of larger size can block the penetration of other substances into plant tissues and mechanically damage the surface through which they enter the cell by creating new and large-size pores. After incorporation in the cells, surplus of NPs might directly interact with internal cellular structures and biomolecules, initiate toxic effects, and generate excessive reactive oxygen species (ROS)^12^. ROS overproduction is responsible for the damage of cell membranes resulting in their increased permeability to greater amounts of NPs^13^. A correlation between NPs bioaccumulation in plant cells and an increase of their concentration in the external environment was observed by Lee et al.^14^. Contrary to other NPs, only a few articles described negative effects of MnNPs. The formation of free radicals as H_2_O_2_, OH· and O_2_·, some of the most toxic ROS, following MnNPs treatment was indicated by Ghosh et al.^15^. ROS can detach hydrogen atoms from fatty acid chains in the lipids that make up cell membranes, resulting in the generation of highly reactive lipid radicals that can further propagate the free radical reaction, causing damage to adjacent lipids. These reactions lead to the formation of lipid peroxides, free radicals and reactive aldehydes such as malondialdehyde (MDA), which further deranges membrane structure and function. Antioxidant enzymes are involved in ROS decomposition, include superoxide dismutase (SOD), which carries out the reaction of conversion of superoxide anion radical into hydrogen peroxide and molecular oxygen. Hydrogen peroxide is then convert with participation of the substrate by peroxidases (POX) or directly by catalases (CAT) into water and oxygen thus preventing of lipid peroxidation^16^.

The authors^15^ reported also that MnNPs at a high concentration were responsible for changes in DNA pattern in a moss *Physcomitrella patens*. Earlier, Pradhan et al.^7^ confirmed MnNPs accumulation in the chloroplast structures after plant exposure to the nanoparticles. The possibility of Mn distribution to chloroplasts was demonstrated also for these metal ions^6^. The presence of MnNPs in these organelles suggests similar mechanisms of their transport as for Mn ions, that is via xylem and phloem for long-distance communication from root to aerial part of plants^17^. Despite a lack of toxic effects in plants, MnNPs accumulation in their tissues may pose a threat to the animals and humans consuming plant products.

The presented experiments focused on the reaction of two wheat cultivars to the incorporation of manganese oxide nanoparticles into their cells following a foliar application. Such a form of MnNPs treatment was rarely described, and our data can provide new information about a direct action of these NPs accumulated trough leaf surface. The need to study the possibility of NPs penetration through the leaves results from a growing content of MnNPs in the air as an effect of increasing dust emission, mainly during the production of steel, iron, and coal combustion^18^. Wheat cultivars were selected based on previous work that showed different responses of these cultivars to the excessive presence of Mn ions^19^. The aims of the experiments was to check: (i) the possibility of Mn accumulation in the leaf cells after MnNPs application; (ii) whether NPs penetration through the leaves may initiate oxidative stress in the cells, as observed during NPs absorption through the root system; (iii) to what extent the membrane structure, particularly membrane lipids of the leaf cells, were modified by the nanoparticles; (iv) whether the physicochemical properties of the chloroplasts can be changed during NPs action.

## Results

Observation of the leaf surface during five days after MnNPs application did not indicate any drastic visual changes (chlorosis, necrosis). However, both treatments reduced leaf fresh weight (Table 1).

**Table 1:** The fresh weight [g], Mn content [mg∙g^−1^], electrolyte leakage [%], MDA concentration [µmol∙ g^−1^ FW] and activity of SOD [U∙mg^−1^ proteins], CAT [U∙mg^−1^ proteins] and POX [U∙mg^−1^ proteins], in the leaves of cvs. Alibi and Nimfa in control plants and after treatment with 125 and 250 MnNPs.

Treatment	FW [g]	Mn content [mg∙g^−1^]	EL [%]	SOD [U∙mg^−1^ proteins]	CAT [U∙mg^−1^ proteins]	POX [U∙mg^−1^ proteins]	MDA [µmol∙ g^−1^ FW]
*Alibi*							
Control	0.58 ± 0.02^a^	0.106 ± 0.010^c^	6.37 ± 0.15^c^	9.93 ± 0.09∙10^−2^	2.44 ± 0.05∙10^−4c^	3.83 ± 0.05∙10^−2c^	1.24 ± 0.05∙10^−2b^
125 NPs	0.56 ± 0.01^a^	2.298 ± 0.016^b^	7.21 ± 0.12^b^	12.60 ± 0.09∙10^−2b^	2.65 ± 0.08∙10^−4b^	4.28 ± 0.04∙10^−2b^	1.44 ± 0.03∙10^−2a^
250 NPs	0.53 ± 0.01^b^	4.541 ± 0.014^a^	8.30 ± 0.13^a^	13.81 ± 0.02∙10^−2a^	6.28 ± 0.10∙10^−4a^	5.63 ± 0.06∙10^−2a^	1.43 ± 0.05∙10^−2a^
*Nimfa*							
Control	0.59 ± 0.02^A^	0.111 ± 0.011^C^	6.15 ± 0.10^C^	9.58 ± 0.05∙10^−2C^	6.29 ± 0.09∙10^−4C^	6.31 ± 0.03∙10^−2C^	1.11 ± 0.02∙10^−2C^
125 NPs	0.58 ± 0.02^A^	2.009 ± 0.018^B^	6.78 ± 0.12^B^	11.09 ± 0.02∙10^−2B^	7.35 ± 0.11∙10^−4B^	6.85 ± 0.02∙10^−2B^	1.17 ± 0.03∙10^−2B^
250 NPs	0.57 ± 0.01^A^	3.649 ± 0.013^A^	7.17 ± 0.15^A^	11.72 ± 0.06∙10^−2A^	7.86 ± 0.11∙10^−4A^	12.01 ± 0.03∙10^−2A^	1.28 ± 0.07∙10^−2A^

Capital letters indicate statistical differences between treatments for Nimfa cultivar, lower case letters for Alibi cultivar. The same letters indicate not significant differences between treatments registered separately for each wheat genotype (p ≤ 0.05).

The decrease was greater for cv. Alibi (about 9%) than for cv. Nimfa, especially at 250 mg/mL NPs, even though fresh weight of both cultivars was similar in control plants. The control plants exhibited similar Mn content. The administration of MnNPs at the lower concentration resulted in an approx. 20-fold increase in Mn level in the leaves of both cultivars. Intercultivar differences occurred at the dose of 250 mg/mL, where a much greater increase in Mn content was observed in cv. Alibi than in cv. Nimfa. However, measurements of the electrolyte leakage did not show any significant differences between the control objects (about 6%) and those treated with either NPs dose (about 8%).

As far as SOD activity was concerned, under control conditions it was higher in cv. Alibi by approx. 3,5% (Table 1). Following the application of 125 NPs, a significant increase in SOD activity was noted, and it was greater for cv. Alibi, by about 10%. The dose of 250 mg/mL NPs only slightly elevated the enzyme activity (about 5%), as compared with lower NPs dose in cv. Nimfa. For cv. Alibi, a continuous 13% enhance was registered. An analysis of CAT and POX activity revealed that in control plants the enzymes were less active in cv. Alibi than cv. Nimfa. For CAT, a greater increase in activity occurred at the lower NPs dose in cv. Nimfa (about 16%), and the higher NPs dose for cv. Alibi (over twice). A reverse dependence was observed for POX, where particularly strong activity (approx. 90%) was demonstrated in cv. Nimfa at the higher NPs dose. Malondialdehyde (MDA) concentration was lower by 10% in cv. Nimfa than in cv. Alibi in control samples, and increased after MnNPs administration (Table 1). In cv. Alibi, this similar 15% rise was already visible at 125 NPs, while in cv. Nimfa it was detected at the higher NPs dose.

**Figure 1:**
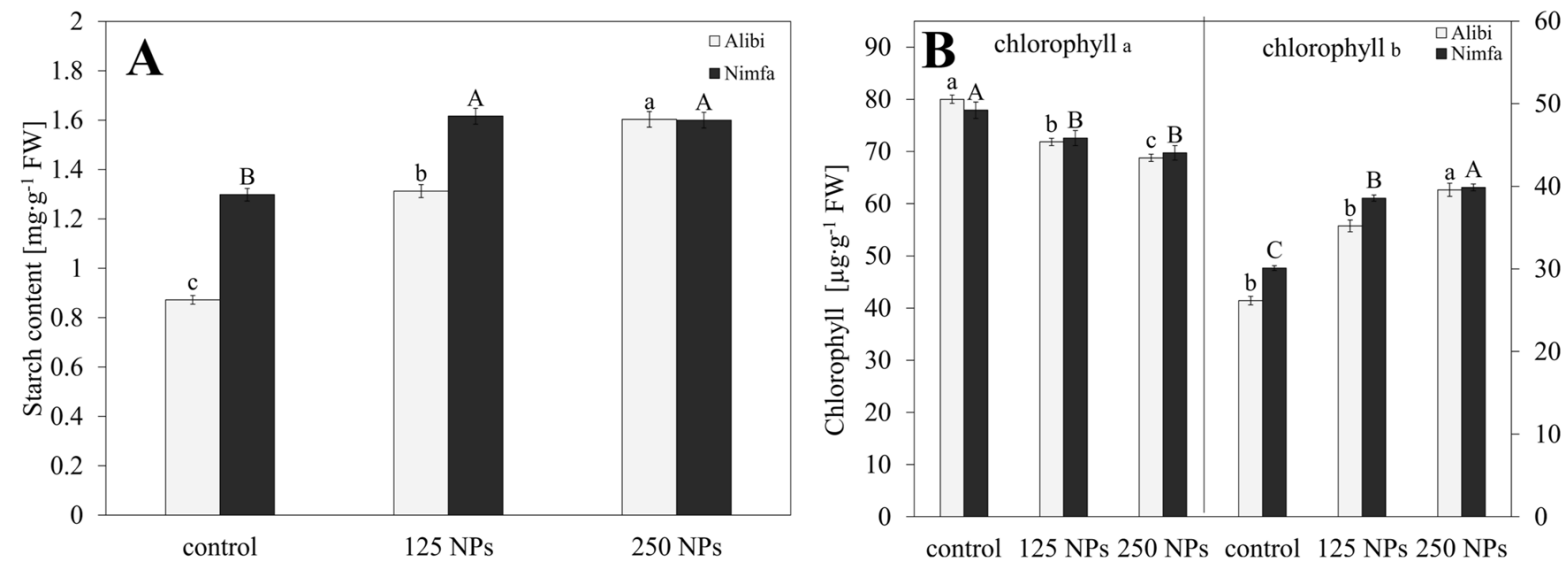
Content of starch [mg∙g^-1^ FW] (**A**); chlorophyll *a* and *b* [µg∙g^−1^ FW] (**B**) in the leaves of cvs. Alibi and Nimfa in control plants and after treatment with 125 and 250 MnNPs. Data are averages from three independent biological replications ± SE. Capital letters indicate statistical differences between treatments for cv. Nimfa, and lower case letters for cv. Alibi. The same letters indicate not significant differences between treatments (*p* ≤ 0.05).

The content of starch enhanced with an increase in the applied of Mn NPs dose in cv. Alibi, by 44 and 77% respectively (Fig. 1A). A similar, about 30% rise in starch content was detected in cv. Nimfa plants exposed to 125 and 250 NPs. The analysis of chlorophyll content revealed that the level of chlorophyll *a* decreased with NPs dose rise in both cultivars, by 5 and 4% at 125 NPs and 15 and 12% at 250 NPs for the cv. Alibi and cv. Nimfa, respectively (Fig. 1B). A reverse dependence was shown for chlorophyll *b*.

**Figure 2:**
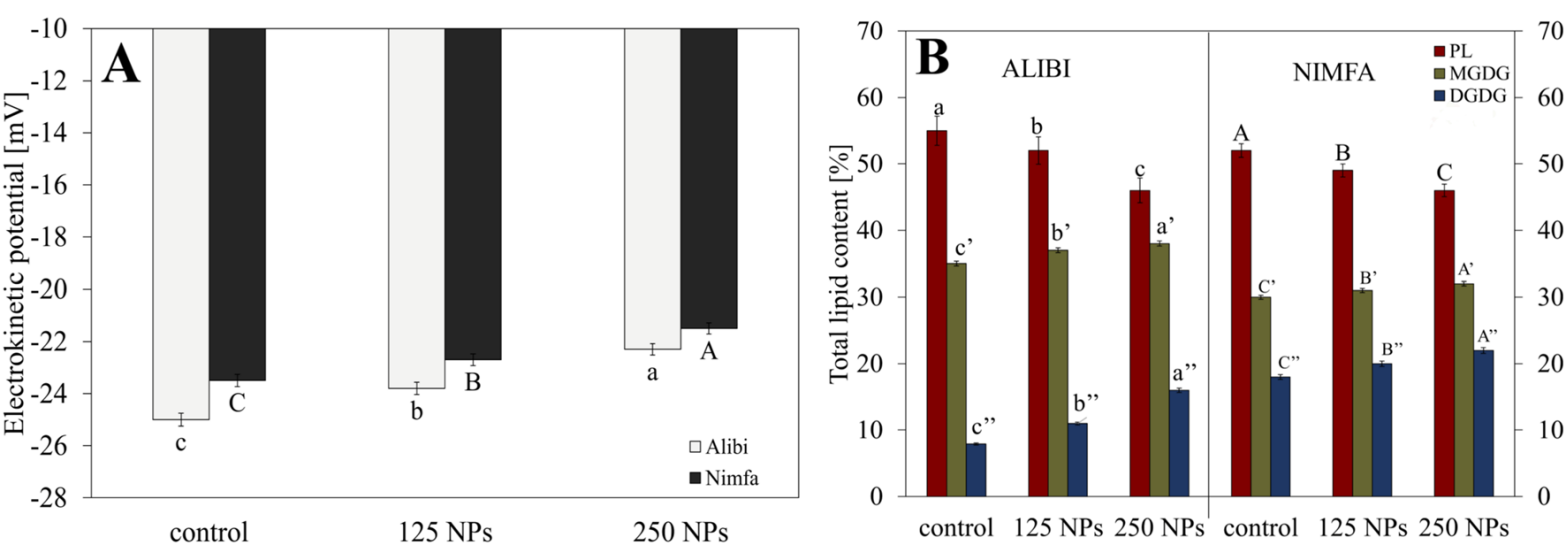
Electrokinetic potential [mV] (**A**); and total lipid content [%] (**B**) in the leaves of cvs. Alibi and Nimfa in control plants and after treatment with 125 and 250 MnNPs. Data are averages from three independent biological replications ± SE. Capital letters indicate statistical differences between treatments for cv. Nimfa, and lower case letters for cv. Alibi. In Fig. 2B statistical significance is marked as A, B, C for cv. Nimfa, and a, b, c for cv. Alibi for PL fraction; A', B', C ' and a', b', c' for MGDG fraction; and A", B", C" and a", b", c" for DGDG fraction. The same letters indicate not significant differences between treatments (*p* ≤ 0.05).

Changes in the electrokinetic potential of the membrane surface in the chloroplasts isolated from the leaves of the studied cultivars were shown in Fig. 2A. Control chloroplasts showed higher electrokinetic potential (by about 6%) in cv. Nimfa than in cv. Alibi. The application of 125 NPs to the leaf surface enhanced the potential by approx.. 5%, in a dose dependent manner. At the higher NPs dose cv. Alibi demonstrated significantly greater (11%) changes in the potential than cv. Nimfa (7%).

Figure 2B presents total lipids isolated from the leaves of both cultivars, separated into fractions of phospho- and galactolipids. In both genotypes, the increase in NPs concentration was accompanied by a raise in digalactosyldiacylglycerols (DGDG) and monogalactosyldiacylglycerols (MGDG) fractions at the expense of phospholipid (PL) fraction. This effect was especially visible for cv. Alibi.

**Figure 3:**
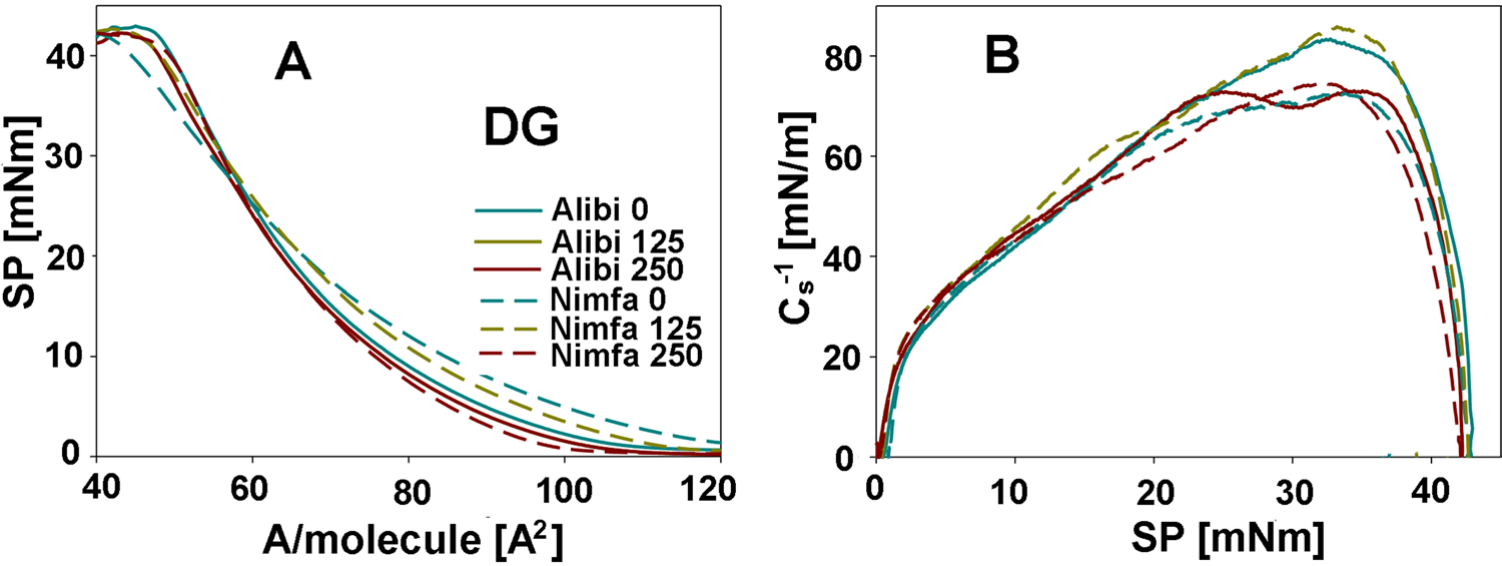
Exemplary Langmuir isotherms (surface pressure π vs area per molecule A) and the dependencies Cs^−1^ versus π corresponding to these systems for the monolayers of the galactolipids—DGDG that were obtained from the leaves of cvs. Alibi and Nimfa in control plants and after treatment with 125 and 250 MnNPs. SP- the value of the surface pressure at the collapse of the monolayer, A/molecule [A^2^]—the area occupied by a single molecule in the maximum packing layer, π_coll_—the value of the surface pressure at the collapse of the monolayer, Cs^−1^—compression modulus.

**Figure 4:**
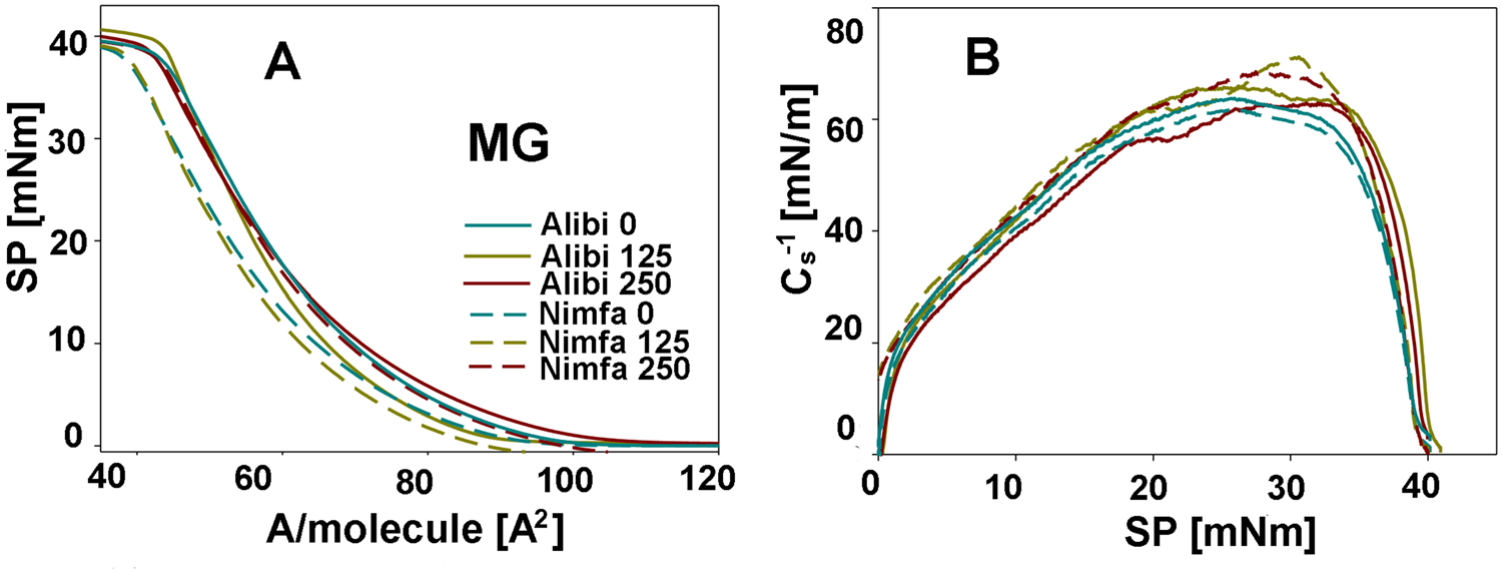
Exemplary Langmuir isotherms (surface pressure π vs area per molecule A) and the dependencies Cs^−1^ versus π corresponding to these systems for the monolayers of the galactolipids MGDG that were obtained from the leaves of cvs. Alibi and Nimfa in control plants and after treatment with 125 and 250 MnNPs. SP- the value of the surface pressure at the collapse of the monolayer, A/molecule [A^2^]—the area occupied by a single molecule in the maximum packing layer, π_coll_—the value of the surface pressure at the collapse of the monolayer, Cs^−1^—compression modulus.

**Figure 5:**
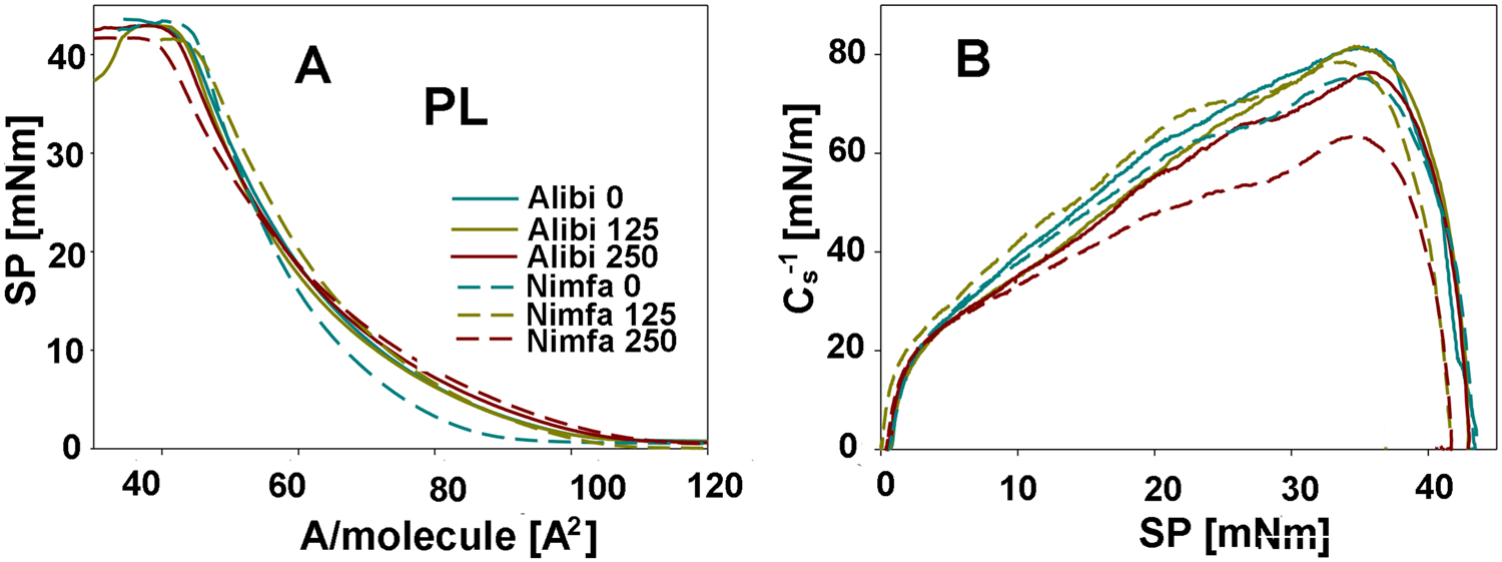
Exemplary Langmuir isotherms (surface pressure π vs area per molecule A) and the dependencies Cs^−1^ versus π corresponding to these systems for the monolayers of the phospholipids—PL that were obtained from the leaves of cvs. Alibi and Nimfa in control plants and after treatment with 125 and 250 MnNPs. SP- the value of the surface pressure at the collapse of the monolayer, A/molecule [A^2^]—the area occupied by a single molecule in the maximum packing layer, π_coll_—the value of the surface pressure at the collapse of the monolayer, Cs^−1^—compression modulus.

Structural properties of membrane lipids were examined individually in DGDG, MGDG, and PL fractions. Examples of adsorption isotherms from which the parameters characterizing membranes: Al_im_—the area occupied by a single molecule in the maximum packing layer; π_coll_—the value of the surface pressure at the collapse of the monolayer and C_s_^−1^—compression modulus were calculated are shown in Figs. 3, 4 and 5. For DGDG and MGDG fractions, A_lim_ was reduced with the increase of NPs dose in both cultivars, and it was particularly visible for cv. Nimfa in DGDG and for cv. Alibi in MGDG fractions, by 13 and 8% respectively (Table 2). The value of π parameter was similar in both cultivars for MGDG fractions, while for DGDG fractions decrease was observed in the cv. Nimfa vs cv. Alibi, by 7 and 2% respectively. Changes in Cs^-1^ parameter indicated an 14% increase in both galactolipid fractions in cv. Nimfa and a decrease in cv. Alibi at growing NPs dose, by 37 and 5% in DGDG and MGDG fractions, respectively. For PL fraction, an 14% elevation in A_lim_ was found, especially for cv. Nimfa, along with increasing concentration of NPs. The value of π showed no meaningful changes in this fraction, regardless of the cultivar and NPs dose. Greater changes were observed for Cs^-1^ parameters that dropped especially at the higher concentration of NPs, by 7 and 18% for cv. Alibi and cv. Nimfa, respectively.

**Table 2:** The effect of manganese nanoparticles on the surface parameters of galactolipid (DGDG, MGDG) and phospholipids (PL) monolayers obtained from the leaves of cvs. Alibi and Nimfa in control plants and after treatment with 125 and 250 MnNPs.

Lipid	A_lim_ [Å^2^]	Π_coll_ [mN∙m^−1^]	C_s_^−1^ [mN∙m^−1^]
DG			
AK	73.3 ± 0.6^a^	42.4 ± 0.6^a^	82.7 ± 0.9^a^
A125	71.5 ± 0.5^b^	42.3 ± 0.5^a^	72.9 ± 0.8^b^
A250	70.5 ± 0.7^c^	39.8 ± 0.7^b^	52.1 ± 0.7^c^
NK	82.0 ± 0.8^A^	41.8 ± 0.4^A^	72.7 ± 0.8^C^
N125	79.9 ± 0.9^A^	41.9 ± 0.6^A^	75.4 ± 0.9^B^
N250	71.2 ± 0.6^B^	41.1 ± 0.5^B^	83.6 ± 0.7^A^
MG			
AK	69.4 ± 0.7^a^	38.5 ± 0.7^a^	65.9 ± 0.8^a^
A125	66.3 ± 0.6^b^	39.2 ± 0.6^a^	63.9 ± 0.9^b^
A250	63.6 ± 0.5^c^	38.9 ± 0.4^a^	62.4 ± 0.8^c^
NK	63.1 ± 0.4^B^	38.1 ± 0.7^A^	62.2 ± 0.7^B^
N125	62.3 ± 0.5^B^	38.7 ± 0.6^A^	61.8 ± 0.9^B^
N250	66.7 ± 0.7^A^	38.6 ± 0.4^A^	71.4 ± 0.7^A^
PL			
AK	67.1 ± 0.4^b^	41.3 ± 0.5^a^	81.7 ± 0.5^a^
A125	66.7 ± 0.5^b^	42.2 ± 0.5^a^	81.6 ± 0.6^a^
A250	68.9 ± 0.4^a^	42.0 ± 0.6^a^	76.1 ± 0.4^b^
NK	61.3 ± 0.6^B^	40.4 ± 0.8^A^	77.0 ± 0.7^A^
N125	69.7 ± 0.5^A^	39.7 ± 0.7^A^	76.4 ± 0.5^A^
N250	70.2 ± 0.6^A^	39.8 ± 0.4^A^	63.3 ± 0.8^B^

*Alim* the limiting area per one molecule, *πcoll* collapse pressure, *Cs*^*−1*^ maximal values of compression modulus.

Capital letters indicate statistical differences between treatments for cv. Nimfa, and lower case letters for cv. Alibi. Mean values (± SE) marked with the same letters are not different according to Students's test (P ≤ 0.05).

## Discussion

Visual symptoms of manganese stress include reduced fresh weight, as well as chlorosis and necrotic spots on the leaves^5,20^. In the tested cultivars, no significant color changes were observed on the leaf surface following MnNPs application, but the decrease of the seedling fresh weight suggested that the applied NPs concentrations, especially the higher one (250 mg/mL), may initiate some toxic reactions. However, these stress effects of NPs did not result from mechanical damage to the leaf tissues, as evidenced by a lack of differences in electrolyte leakage between the control and treated objects. A rise in electrolyte leakage is usually indicated as one of the major factors responsible for increased cell membrane permeability of plants growing under different stresses^21^. Relative electrolyte leakage (in comparison with total ion content) exceeding 50% informs of the irreversible damage to cell membranes. In our study, no significant electrolyte leakage was found, and we concluded that MnNPs treatment did not lead to drastic destruction of the plasmalemma.

As shown in spectrometric studies, an increase of Mn level in the leaves by about 20–45 times in relation to the control, suggests the penetration of NPs into the internal structures of the leaf. Interestingly, greater content of Mn (at the highest dose of NPs) was noted for cv. Alibi, for which the administration of Mn ions via the root system to leaves resulted in lower absorption of this element than in cv. Nimfa^19^. A drastic increase in Mn accumulation in the presence of Mn particles was previously demonstrated by Ghosh et al.^16^ in *Physcomitrella patens*. The possibility that the nanoparticles enter the cells via the same transporters as other ions was suggested in many studies. The knowledge on Mn ion transporters is limited, and only a few transporters involved in Mn uptake by roots have been identified up to now^22^. The properties of Mn transporters in the plasma membrane of leaf cells have not been clearly described so far, however, Sasaki et al.,^23^ and Peris-Peris et al.^24^ suggested these transporters exist also in leaf organelles.

The changes we observed in the structural and biochemical properties of leaf membranes may suggest the possibility of MnNPs interactions with lipid fractions of the membranes, which may affect their stiffness and in consequence activation of the transmembrane protein transporters. Fatty acid saturation of the membrane building lipids is the main factor responsible for the membrane stiffness. On the other hand, the changes in lipid saturation were considered the effects of the membrane modification to protect cells against toxicity of stress factors^25^, or the signs of partial destruction of these structures upon stress^26^. One of the determinants of oxidative stress is the increase of MDA concentration, informing of the reduction of fatty acid unsaturation. The rise of lipid saturation is triggered by ROS at the sites of double bonds in unsaturated fatty acids^27^. For the tested cultivars, a progressive increase in MDA levels was observed as NPs doses increased, but the change was not as significant as in the case of other stressors, where even a 20% rise in MDA concentration was reported^28,29^. Therefore, it can be concluded that foliar administration of NPs initiated oxidative stress but the effects of this stress were relatively small, and greater in cv. Alibi than cv. Nimfa. The activation of antioxidant enzymes confirmed the occurrence of stress conditions. SODs, CAT and POX are responsible for the inactivation of excessive ROS^30^. Activation of these enzymes may reduce possible ROS reactions with intracellular biomolecules leading to their destruction. The enhancement of SOD activity following the application of MnNPs could be also due to more abundant presence of Mn in the cells, as shown for MnSOD after treatment with Mn ions^31^.

Higher activity of SODs (enzymes transforming peroxide radical into hydrogen peroxide), found in cv. Alibi than in cv. Nimfa may have suggested greater ROS production in cv. Alibi. However, both CAT and POX, responsible for deactivation of hydrogen peroxide, showed lower activity in cv. Alibi. Such an effect might be related to lower efficiency of H_2_O_2_ removal from the cells of this cultivar and might indirectly indicate its greater sensitivity to stress. Progressive growth of starch content in cv. Alibi initiated by MnNPs application confirmed the occurrence of oxidative stress in this cultivar, exacerbated along with growing concentration of NPs. Enhanced accumulation of starch in response to various stress factors was demonstrated in many studies as a disorder of photosynthesis^19,32^. Interestingly, in cv. Nimfa starch level rise noted at 125 MnNPs was maintained at higher MnNPs dose.

Disorders of photosynthesis were also indicated by a decrease in chlorophyll *a* to *b* ratio. Baldisserotto et al.^33^ suggested that reduced chlorophyll content may be due to Mn oxidation in chloroplasts initiated by excessive production of ROS. Disruption of photosynthesis efficiency and a drop in chlorophyll content under excessive Mn was shown by Liu et al.^34^. In the tested cultivars, the changes in chlorophyll content were rather small, as confirmed by lack of leaf chlorosis. However, they indicated the possibility of disturbances in chloroplast functioning evoked by the presence of the nanoparticles. Experiments conducted on the chloroplasts isolated from the leaves of plants subjected to NPs showed changes in the physicochemical properties of their membranes. An increase of the chloroplast surface charge toward positive was detected by measuring the electrokinetic potential. The potential shifted toward less negative values after NPs treatment. A stronger effect visible for cv. Alibi chloroplasts even at the lower NPs dose indicated greater modifications of cv. Alibi membranes. Such modifications could diminish the affinity of cationic substances to more positively (less negatively) charged surface and reduce the effectiveness of Mn ion absorption. A similar direction of changes in the electrokinetic potential of chloroplast surface was shown under Cd stress^35^.

The modifications in the membrane lipid structure initiated by MnNPs were investigated in detail in the experiments carried out for individual lipid membrane fractions isolated from the leaves. An increase in A_lim_ area per a lipid molecule in the PL (in relation to the control) was due to enhanced fatty acid unsaturation of this fraction and may be interpreted as a factor increasing the membrane fluidity^25^. PLs constitute the main lipid fraction of the plasmalemma and for the tested wheat cultivars they accounted for about 50% of the pool of lipids extracted from the membranes. Enhanced fluidity of PL fraction may lead to greater exposure of the hydrophobic part of the lipids (increasing the distance between polar groups) in order to reduce the possibility of adsorption of ionic components on non-polar parts of the membranes. Such effects initiated by MnNPs treatment, more pronounced in cv. Nimfa, may suggest that in this cultivar the studied NPs activated the protective mechanisms to a higher degree than in cv. Alibi. The changes in membrane stiffness under the treatment with MnNPs were also evidenced by the values of π_col_ and Cs^−1^ parameters, suggesting greater "decompression" of the lipids of PL fraction in cv. Nimfa. The strongest effects (Cs) shown after the administration of 250 mg/mL NPs may indicate that this concentration activated the defense mechanisms to a much greater extent than 125 NPs. For the studied galactolipids, the reduction of A_lim_ values (relative to the control) may have designated an increase in lipid saturation of this fraction, stimulated by NPs treatment. In general, the changes in galactolipids represent the reaction of chloroplast membranes, as these lipids are the main lipid fractions in plastids^36^. The polarity of MGDG and DGDG fractions results mainly from the presence of sugar groups. The decrease of A_lim_, together with the rise of π_col_ and Cs^-1^ upon NPs treatment, represented a trend toward increasing lipid stiffness (A_lim_) and membrane stability (parameters π and Cs^-1^). These effects were especially visible in DGDG fraction of both cultivars, however, some differences between the tested cultivars were ascribed to the extent of changes between MGDG and DGDG fraction. This proved the cultivar specificity in response to NPs, which was also confirmed in the analysis of other biochemical parameters mentioned above. In addition, it indicated that chloroplasts were the organelles that determined the cultivar response to NPs.

## Conclusions

MnNPs were shown to penetrate the leaves, leading to an increase in cellular Mn pool, and the mechanism of Mn uptake through the leaves was different than through the root system. However, the investigated concentrations have initiated stress reactions (generation of excessive ROS) manifested in the activation of antioxidant enzymes. Intercultivar differences in the activation of CAT and POX, responsible for decomposition of hydrogen peroxide, may indicate various sensitivity of the cultivars to MnNPs stress. MnNPs treatment led to photosynthetic disorders, which resulted in an increase in starch concentration and lowered synthesis of chlorophyll. The increase in the value of electrokinetic potential of chloroplast membranes (indicating changes in their polar parts) may reduce the tendency to adsorb Mn cations on their surface, while rebuilding of the hydrophobic parts (galactolipid structure detected by means of Langmuir technique) in the direction of fatty acid saturation can reduce the possibility of incorporating Mn into the chloroplasts. Greater changes shown in cv. Alibi can be associated with greater penetration of Mn into the cells of this cultivar.

## Materials and methods

### Properties of nanoparticles

MnNPs were obtained from US Research Nanomaterials Inc. (Houston, TX USA) as Manganese Oxide Nanoparticles Water Dispersion/Mn_3_O_4_ Nanopowder Water Dispersion (20 wt %, 30 nm), at two oxidation states (II and III), and portrayed as MnOxMn_2_O_3_.

According to the data provided by the company, the MnNPs are characterized by strong absorption and oxidation ability.

### Plant material

Grains of two spring wheat cultivars (Alibi and Nimfa) were received from the Polish Plant Breeding Station in Strzelce. The grains were sterilized with 80% ethanol and 10% perhydrol and then rinsed four times with distilled water. After sterilization, the seeds were germinated in Petri dishes at 20 °C, in the dark. The germinated seeds were planted into the holes of a polystyrene plate placed on a 10 L container filled with 50% Hoagland nutrient solution. The seedlings were grown under relative humidity 45–50%, at light intensity of 800 μmol (photon) m^−2^ s^−1^ (SQS, Hansatech Ltd, Kings Lynn, United Kingdom), and 16 h photoperiod (17/20 °C night/day) for 10 days. MnNPs were applied on the surface of two fully developed leaves at the concentration of 125 and 250 mg/mL (1 mL per plant). The doses were labelled as 125 NPs and 250 mg/mL NPs. The leaves of the control plants were treated with pure water. After 5 days, the seedlings were harvested and the leaves were separated from the roots. Only the aerial parts were selected for analysis. After thorough and multiple washing with deionized water, the tissues were weighed and frozen in liquid nitrogen for further studies. Electrolyte leakage and electrokinetic potential were determined in fresh leaves. Collection of plant material comply with relevant institutional, national, and international guidelines and legislation. Leakage of electrolyte from leaf discs (ϕ = 5 mm) was measured according to the procedure described in details by Filek et al.^37^.

### Determination of manganese concentration

Samples of freeze-dried leaves (0.02 g) (Freeze Dry System/Freezone 4.5, Labconco, USA) were mineralized in ultrapure concentrated nitric acid (Merck, Darmstadt, Germany) in a closed microwave system (Uni Clever, Plazmatronika, Poland). Mn (55) was analyzed by ICP-MS spectrometry (Elan DRC-e, Perkin Elmer, Shelton, USA), according to the procedure described in detail by Tobiasz et al.^38^.

### Antioxidant enzyme assays

Superoxide dismutase (SOD; EC 1.15.1.1) activity was analyzed spectrophotometrically at λ = 550 nm, using a cytochrome method and the protocol described by McCord and Fridovich^39^. The activity of peroxidase (POX; EC 1.11.1.9) was determined according to Luck^40^ protocol, by spectrophotometric measurements (λ = 485 nm) of the products formed during a reaction with 1% p-phenylenediamine in the presence of 0.03 mM H2O2. Catalase (CAT; EC 1.11.16) activity was analyzed at λ = 240 nm using Aebi^41^ procedure (reaction with 0.03 mM hydrogen peroxide). All determinations were carried out on a spectrophotometer (Thermo Fisher Evolution 201/220 UV–Visible Spectrophotometer, Waltham, USA).

### Starch content determination

Starch content was established with anthrone reagent as described in detail by Janeczko et al.^42^. Decomposition of starch into glucose was performed with 0.5 unit of amyloglucosidase (EC 3.2.1.3), and 1 unit of α-amylase (EC 3.2.1.1).

### Determination of chlorophyll content

Chlorophyll content was measured by a cold extraction of leaves with 80% acetone, and the pigment amounts were determined spectrophotometrically at fixed wavelengths (λ = 470 nm, λ = 624 nm, λ = 645 nm, λ = 663 nm, and λ = 730 nm). The concentration of chlorophyll *a* was calculated from Formula (1), and of chlorophyll *b* from Formula (2).

1 $${\text{Chl}}_{a} = { 12}.{67}\left( {{\text{A}}_{{{663}}} - {\text{ A}}_{{{73}0}} } \right) \, {-}{ 2}.{65}\left( {{\text{A}}_{{{645}}} - {\text{A}}_{{{73}0}} } \right) \, {-} \, 0.{29}\left( {{\text{A}}_{{{645}}} - {\text{A}}_{{{73}0}} } \right)$$

2 $${\text{Chl}}_{b} = { 23}.{6}\left( {{\text{A}}_{{{645}}} - {\text{ A}}_{{{73}0}} } \right) \, {-}{ 4}.{23}\left( {{\text{A}}_{{{663}}} - {\text{A}}_{{{73}0}} } \right) \, {-} \, 0.{33}\left( {{\text{A}}_{{{624}}} - {\text{A}}_{{{73}0}} } \right).$$

### Malondialdehyde (MDA) analysis

The leaves (1 g) were homogenized in 0.5% trichloroacetic acid (TCA) and centrifuged at 10,000 g for 10 min (Micro 185, Hettich, Tuttingen, Germany). MDA content was established by a reaction with thiobarbituric acid (TBA; 0.5%), according the protocol presented by Dhindsa et al.^43^.

### Lipid extraction

Lipids were extracted and separated according to the method used by Gzyl-Malcher et al.^44^.

### Analysis of lipid structure parameters

Lipid monolayers were formed by applying chloroform solutions of appropriate lipids to the surface of aqueous solutions. Surface pressure isotherms were recorded by using a Langmuir trough (Minitrough, KSV, Finland) equipped with a Pt-Wilhelm plate for surface tension detection (accuracy ± 0.1 mN/m). The experiments were carried out at 25 ℃. The measurements were repeated three or four times achieving repeatability of 0.1–0.3 Å^2^.

Isotherms, i.e. the dependence of the surface pressure (π) on the surface per lipid molecule (A), were the basis for determining the structural parameters of the monolayers formed, i.e. A_lim_—the area occupied by a single molecule in the maximum packing layer and π_coll_—the value of the surface pressure at the collapse of the monolayer. Moreover, the compression modulus Cs^−1^ = − (dπ/dlnA) was calculated, which indicated the mechanical strength of the layer during compression. This parameter provides information about the flexibility of the layer.

### Electrokinetic potential in chloroplasts

Chloroplasts were isolated according the protocol of Block et al.^45^. Then, the chloroplasts were centrifuged in a Percoll gradient (40%/80%) to obtain completely purified organelles^46^. For determination of their electrokinetic potential, the chloroplasts were re-suspended in the isolation buffer. All procedures were carried out at 0–4 °C.

Electrokinetic potential of freshly isolated chloroplasts was analyzed according to the procedure described by Filek et al.^47^. The measurements were made in solutions (0.1 mM KCl, 0.6 M mannitol) providing constant ionic strength and osmotic pressure. The electrokinetic potential was calculated from electrophoretic mobility data (according to Smoluchowski equation) using Zeta-PLUS apparatus (Brookhaven, USA).

### Statistical analysis of data

Data are presented as means ± SE. The statistical analysis involved Duncan’s multiple-range test, and statistical significance was estimated at p < 0.05 using PC SAS 8.0 software (Tulsa, USA). Significance of the means was evaluated with SAS ANOVA.

## Data Availability

The datasets used and/or analysed during the current study available from the corresponding author on reasonable request.

## Abbreviations

MGDG: Monogalactosyldiacylglycerol
DGDG: Digalactosyldiacylglycerol
PL: Phospholipids
SOD: Superoxide dismutases
POX: Peroxidases
CAT: Catalase
A_lim_: The area occupied by a single molecule in the maximum packing layer,
π_coll_: The value of the surface pressure at the collapse of the monolayer
Cs^−^^1^: Compression modulus
